# Bone healing response to a synthetic calcium sulfate/β-tricalcium phosphate graft material in a sheep vertebral body defect model

**DOI:** 10.1002/jbm.b.32758

**Published:** 2012-07-30

**Authors:** H L Yang, X S Zhu, L Chen, C M Chen, D C Mangham, L A Coulton, S S Aiken

**Affiliations:** 1 Department of Orthopaedic Surgery, The First Affiliated Hospital of Soochow UniversitySuzhou, China 215006; 2 Department of Musculoskeletal Pathology, Robert Jones & Agnes Hunt Orthopaedic Hospital NHS TrustOswestry, Shropshire, UK; 3 Department of Human Metabolism, Academic Unit of Bone Biology, University of Sheffield Medical SchoolSheffield, UK; 4 Department of Research and DevelopmentBiocomposites, Staffordshire, UK

**Keywords:** kyphoplasty, vertebroplasty, calcium sulfate, β-tricalcium phosphate, bone cement, PMMA

## Abstract

The introduction of a material able to promote osteogenesis and remodelling activity in a clinically relevant time frame in vertebroplasty and kyphoplasty procedures may have patient benefit. We report the *in-vivo* performance of a biphasic synthetic bone graft material (Genex Paste, Biocomposites, UK) [test material], composed of calcium sulfate and β-tricalcium phosphate, implanted into a sheep vertebral defect model. Cavities drilled into 4 adjacent vertebrae (L2 to L5) of 24 skeletally mature sheep were; (1) filled with the test material; (2) filled with commercially available polymethylmethacrylate [PMMA] cement; (3) remained empty [sham]. Analysis was performed immediately after implantation and at 8, 16, and 36 weeks post implantation. Sites were evaluated for bone growth with microCT analysis, histological examination, and mechanical testing under compression. The test material exhibited an improved tissue response over the PMMA, indicating a superior biological tolerance. MicroCT and histology indicated marked osteoregenerative capacity of the test material when compared with sham and the PMMA. The percentage of new bone formation was higher for the test material than sham at 16 and 36 weeks post implantation, with bone regeneration almost complete at 36 weeks in this group. Resorption of test material and the integration into new bone tissue were demonstrated. © 2012 Wiley Periodicals, Inc. J Biomed Mater Res Part B: Appl Biomater, 2012.

## INTRODUCTION

Vertebral body augmentation is widely used to treat fractures of the vertebral body due to trauma, osteoporosis, or other lytic conditions. Since the mid-1980s,^1^ the most commonly used treatment for vertebral body augmentation involves the introduction of polymethylmethacrylate (PMMA) bone cements into the vertebral body.

PMMA cement offers several advantages to the orthopaedic surgeon, including biomechanical strength and stiffness.^2^ However, it possesses inherent disadvantages including a lack of remodelling potential.^3^ In addition PMMA has excessive stiffness, potential monomer toxicity,^4^–^6^ and high polymer curing temperatures.^7^ Research in recent years has led to the development of a number of synthetic, ceramic based bone cements which show potential for vertebral body augmentation.^7^–^9^ These materials exhibit the capability to resorb over time, the potential for replacement with new bone and could avoid the application of PMMA cements. However, few have been evaluated in a vertebral defect model^10^. The study presented here evaluates the performance of the test material (Genex Paste, Biocomposites, UK) in a sheep vertebral body defect model. This test material consists of calcium sulfate and β-tricalcium phosphate (β-TCP), in a weight ratio of 1:1. It is synthetic and designed to be fully resorbed. Applied as an injectable paste to the surgical site, it sets *in-situ* at body temperature within 15 minutes.^11^ The setting is due to the calcium sulfate hemihydrate component present in the supplied powder which reacts when combined with a supplied volume of water.

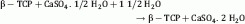


The hemihydrate is converted to calcium sulfate dihydrate that sets hard within minutes of mixing.

### Study aims

The study evaluates the performance of the test material in a vertebral body defect with respect to new bone growth, host tissue response, and resorption. The performance was compared to medical grade PMMA cement (The Tianjin Composition Material Research Institute, China), and an empty sham defect. The application of PMMA is commonplace in the treatment of vertebral fractures, and as such, the comparative behaviour of the test material is important to determine any potential as an alternative implant material.

## MATERIALS AND METHODS

The materials were evaluated in a vertebral body defect using 24 skeletally mature sheep (2.5 years old ± 0.5, body weight 40–50 kg). Ethics approval was obtained through the animal care and ethics committee of the First Affiliated Hospital of Soochow University. Animals were cared for in accordance with guidelines “GB14925-2010. Laboratory animal- Requirements of environment and housing facilities”. Each surgical site was shaved 24 h prior to surgery. Following intravenous anaesthesia, a longitudinal incision was made, followed by passive separation of the peritoneum and muscle, and exposure of the lumbar vertebrae, L2 to L5. For each vertebra a 2.0 mm diameter guide hole was drilled equidistant between the endplates, down into the vertebra sagittal plane to a depth of 9 mm. Larger drill bits (4.0 mm then 6.0 mm) were used to expand the defect diameter, maintaining a depth of 9 mm. Bone debris was removed. The resultant vertebral defects in each animal were randomly assigned treatment:

1. Two defects were filled with the test material, prepared in accordance with the manufacturer's instructions.^11^
2. One defect was filled with PMMA cement prepared in accordance with the manufacturer's instructions.
3. One sham defect remained unfilled.

The test material and the PMMA were allowed to set (15 min) and the region was flushed with saline. The sites were then closed in layers. Penicillin (160 mg unit/day, IM), was administered for 5 days post-operatively. Implant harvesting was conducted 0, 8, 16, and 36 weeks postoperatively. At each point, six sheep were randomly selected and euthanized. The implanted vertebrae at each time point were explanted and analysed by microCT and histological analysis as described in Tables I and II.

**Table I:** MicroCT Analysis Allocation of Explanted Vertebrae

Time Point	Sham Group	PMMA Group	Test Material Group
0 weeks	2	2	2
8 weeks	2	2	2
16 weeks	2	2	3
36 weeks	2	2	2

**Table II:** Analysis Allocation of Explanted Vertebrae at Each Time Point

	Sham Group	PMMA Group	Test Material Group
Mechanical testing	2	2	3
Decalcified histology, paraffin embedding	2	2	5
Undecalcified histology, resin embedding	2	2	4
Total number at each time point	6	6	12

Total number analyzed at each time point was 24. Therefore the total number of explanted and analysed vertebrae for all 4 time points was 96.

Selected explanted vertebrae from each time point were evaluated by means of micro-CT to determine the amount and nature of bone regeneration. As this analysis technique is nondestructive, it was performed prior to histological processing. The number of vertebrae analysed by MicroCT at each time point is shown in Table I. At each time point, all of the explanted vertebrae (24 vertebrae x 4 time points) were subsequently analysed as shown in Table II.

### MicroCT analysis

On explanation, vertebrae for microCT analysis were immediately fixed in neutral buffered formalin (10%). The vertebra were scanned using a microCT scanner (model 1076, Skyscan, Belgium) at 50 kV, 200 μA and a 0.5 mm aluminium filter. The pixel size was 18.26 μm. Two images were captured every 0.7° through 180° rotation of the sample, the exposure time per image was 420 ms. The x-ray images were reconstructed using Skyscan NRecon software, set with a dynamic range of 0–0.15 and analyzed using Skyscan CT analysis software. Implant volume was determined where applicable, and trabecular bone analysis performed; rendered three-dimensional models were also constructed.

### Histological analysis

#### Decalcified histology

Vertebrae assigned to decalcified histological analyses were soaked in neutral buffered formalin (10%) for a minimum of 48 h on explantation, and then decalcified in 9% formic acid solution and rinsed on completion with water. Vertebrae from the test material or sham group were then immediately processed to paraffin wax. Vertebrae from the PMMA group were processed in chloroform and xylene to dissolve the PMMA material, and then processed to paraffin wax. The decalcified, embedded vertebrae were sectioned parasagittally (5 μm) to produce slides with a circular cross section through the implantation area, mounted and stained (haematoxylin and eosin [H&E]).

#### Undecalcified histology

On explantation, vertebrae assigned undecalcified histological analysis were dehydrated in sequential solutions of ethanol (75, 85, 95, and 100%), and then processed to methyl methacrylate embedding. The undecalcified, embedded vertebrae were sectioned in a parasagittal orientation, as described above. Sections were cut to a thickness of 100 μm using a saw microtome (LEICA SP1600, Germany), and then ground and polished to a thickness of 50 μm and stained (picric acid).

The stained sections, decalcified and undecalcified, were examined under light microscopy (Olympus BX51), to qualitatively assess the bone response. Images of the histology were digitally captured (Zeiss AxioScope optical microscope, with Axiocam ICcl colour camera, AxioVision software v4.7).

The histology data were used to determine the following primary and secondary endpoints of the study; (1) Assessing the host tissue response to the test implant material compared with the sham and PMMA groups; (2) *In-viv*o resorption of the test material.

The *in-vivo* resorption of the test material and the formation of new bone were evaluated by means of Histomorphometric assessment and compared to sham and PMMA sites. A series of microscope images of the implant area were combined to a composite image (RasterStich, VextraSoft, Version 1.80) of the drilled void and area implanted with graft material/cement. Adobe Photoshop Elements (Adobe, Version 5.0) was then used to delineate the area of graft material and new calcified bone tissue present in the image which was then converted to a black/white (binary) view. Using an image-processing program (ImageJ, National Institutes of Health, USA, Version 1.38x), the remaining, unresorbed, graft material, and new calcified bone tissue was converted to a percent of amount in each surgically created defect. This was performed for two histology samples at 8, 16, and 36 weeks for each of the treatment groups.

### Mechanical testing analysis

Vertebrae assigned for mechanical testing were soaked in neutral buffered formalin (10%) on explantation. Mechanical testing was performed as described by Tohmeh et al.^12^ The concave of the endplates of each vertebra were filled with PMMA such that a compressive load could be applied across parallel surfaces. Samples were loaded in a mechanical testing rig (Instron 5566R, UK) and a compressive load applied. The vertebrae were pre loaded at 200N for 30 s, and the loading increased by displacement at 5 mm/min. Instrumentation recorded at 10 Hz frequency and measured load—displacement curves. Each vertebra was tested only once since the mechanical testing was destructive. The peak compressive load and stiffness was determined for all samples. The highest point of the graph was taken as the peak compressive load. The stiffness was taken in the linear region of the curve based on a linear regression. Mechanical testing determined the compressive strength of the vertebrae implanted with the test material compared to the sham and PMMA groups. Statistically, the differences between each postoperative time were compared using the one-factor analysis of variance with the post hoc Tukey–Kramer test for multiple post hoc group comparisons.

## RESULTS

All animals were revived from surgery within 4 to 6 h, and fully recovered within 2 weeks. The surgical sites were well healed.

### Gross observations

Explanted samples indicated no obvious inflammation of soft tissue. Vertebrae from the 8-week time point showed some differences; for vertebrae from the test material group, the drill hole had become completely covered with new cortical bone and no test material was evident. In vertebrae from the sham and PMMA groups, the drill hole was only partially covered with new cortical bone. Vertebrae from the 16 and 36 week time points indicated no visual difference between the implanted groups, with all drill holes completely covered with cortical bone.

### Micro-CT analysis

**Figure 1:**
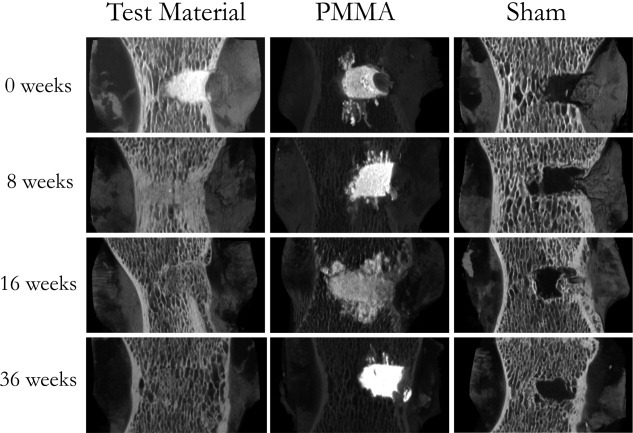
Rendered 3D models sliced to show the cavity at 0, 8, 16, and 36 weeks.

3D reconstructed data sets were generated from the microCT scans to evaluate bone regeneration in the sites. Sliced images from the rendered 3D models of the vertebrae demonstrated differences between the implanted and sham sites (Figure 1).

Three dimensional analysis indicated that the sites implanted with test material demonstrated the largest degree of bone healing. No new bone was evident within areas implanted with PMMA, as a result of its nonresorbable nature. However, there was some evidence of new bone formation over the site of implantation, likely as a result of periosteal bone growth. The extent of migration of the PMMA within adjacent trabecular bone was evident. The sham sites demonstrated some new bone formation within the defects, but to a lesser degree than that observed in the test material group.

**Figure 2:**
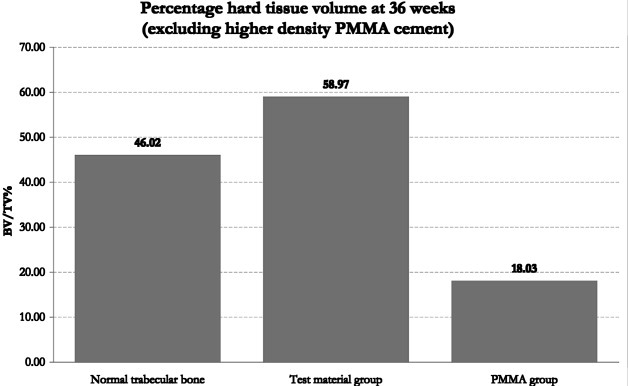
Percentage radiopaque tissue volume at 36 weeks, excluding higher density PMMA cement.

**Figure 3:**
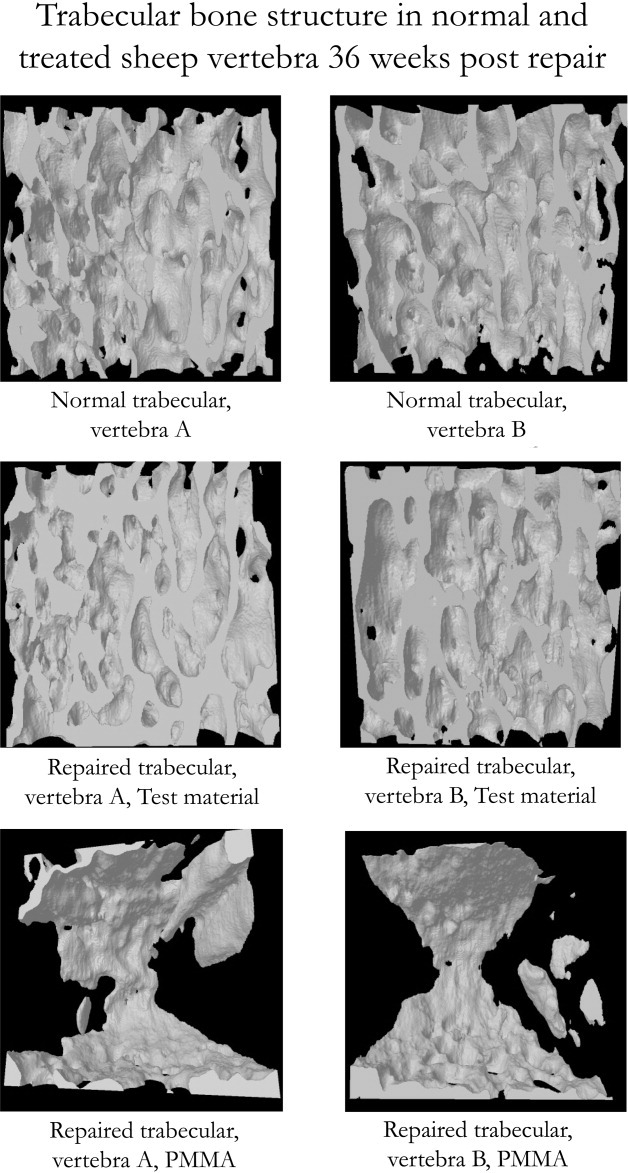
Sliced three dimensional rendered models of normal bone trabeculae and regions repaired with Test material and PMMA.

Quantitative analysis of new bone formation was performed on three dimensional reconstructed data. Because of the higher X-ray absorption of the PMMA cement, a result of the radiopaque barium sulfate component, it was possible to analyse the data excluding the higher density cement, which was most of the PMMA. The test material possessed a higher radiopacity on placement, as a result of the calcium sulfate component. However, at subsequent time points it was not possible to isolate the test material from the bone, as residual material possessed radiodensity within the range of cortical and cancellous bone. An analysis of percentage radiopaque tissue volume in the test material and PMMA implant sites (excluding the higher density PMMA cement) at 36 weeks, compared with normal trabecular bone in the vertebrae is shown in Figures 2 and 3.

The observed volume of radiopaque tissue was higher for the test material sites than equivalent normal sites. However, the contribution to this result from residual test material cannot be determined due to reasons discussed previously. The lowest quantity of bony tissue (excluding PMMA cement) at 36 weeks was observed in the PMMA group. At 36 weeks, the test material vertebrae demonstrated a trabecular structure similar to that of the normal vertebral trabecular bone, whereas the PMMA implanted vertebrae did not.

**Figure 4:**
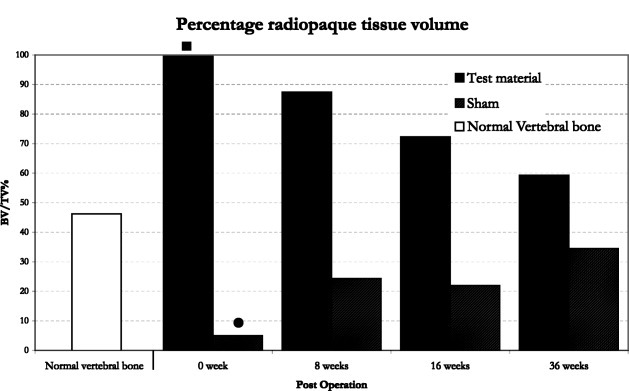
Percentage radiopaque tissue volume for Test material and Sham sites, compared to that observed in normal vertebral trabecular bone. ▪ High value at 0 week indicates test material filled sites and not radiopaque tissues. ● Low value refers to empty sham sites.

**Figure 5:**
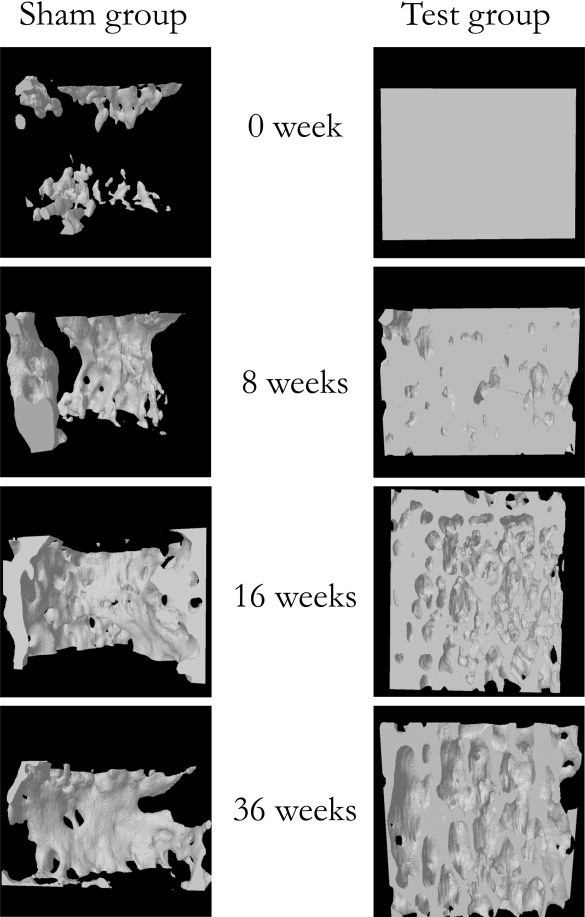
Sliced three dimensional rendered models of the sham group and regions repaired with Test material.

As the PMMA implanted sites have limited scope for normal bone regeneration, the comparison in structural development between the test material and sham sites were conducted. An analysis of percentage radiopaque tissue volume in the test material and sham implant sites at 0, 8, 16, and 36 weeks, is shown in Figures 4 and 5. The high radiopaque tissue volume for the test group (99.5%) at 0 weeks reflects the fact that the site was completely filled with test material. Subsequent sacrifice points demonstrated a gradual decrease in the radiopaque tissue volume. Conversely, in the sham group, following the low initial volume as a result of the drilling procedure, a gradual increase in the radiopaque tissue volume present was observed. Neither test material nor sham achieved the radiopaque tissue volume measured in normal vertebral bone at 36 weeks. These measurements are reflected in the sliced three dimensional rendered models. The test material group fills the site at 0 weeks, and the radiopaque tissue structure gradually opens up over the 36-week period. In the sham group we see a gradual infill by radiopaque tissue starting to take place.

**Figure 6:**
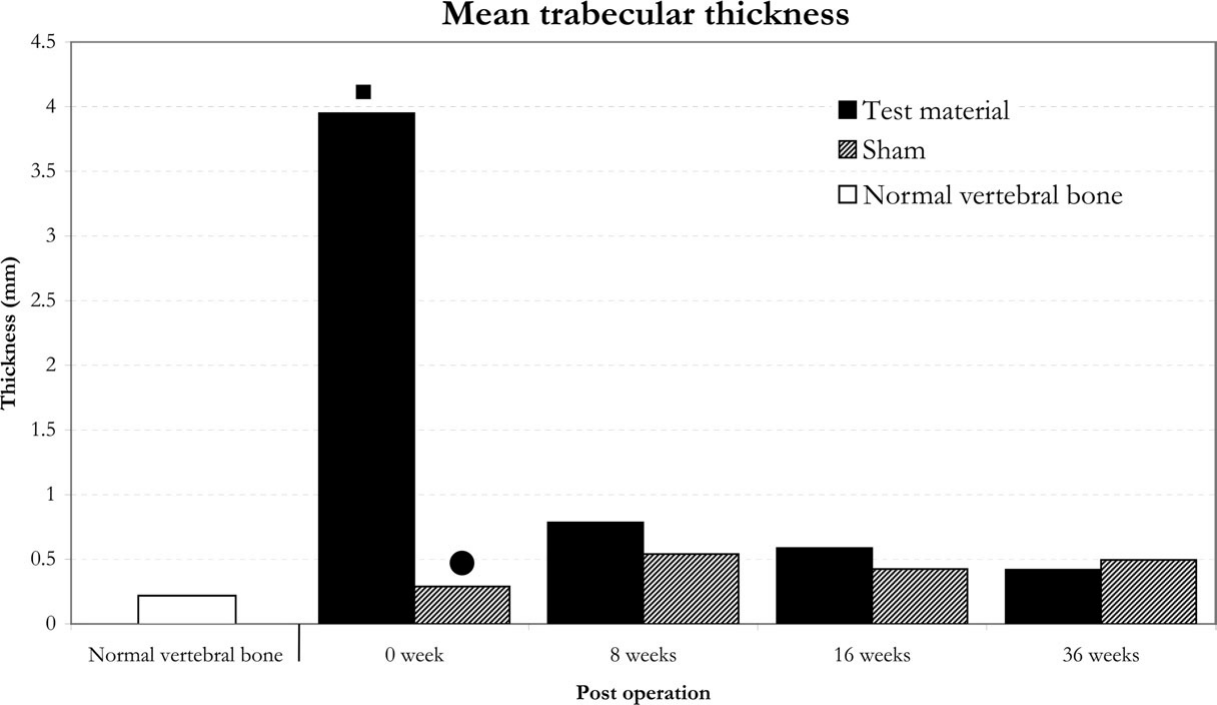
Mean trabecular thickness for Test material and Sham sites, compared to that observed in normal vertebral trabecular bone. ▪ High value at 0 week indicates test material filled sites and not bone trabeculae thickness. ● Low value refers to empty sham sites.

**Figure 7:**
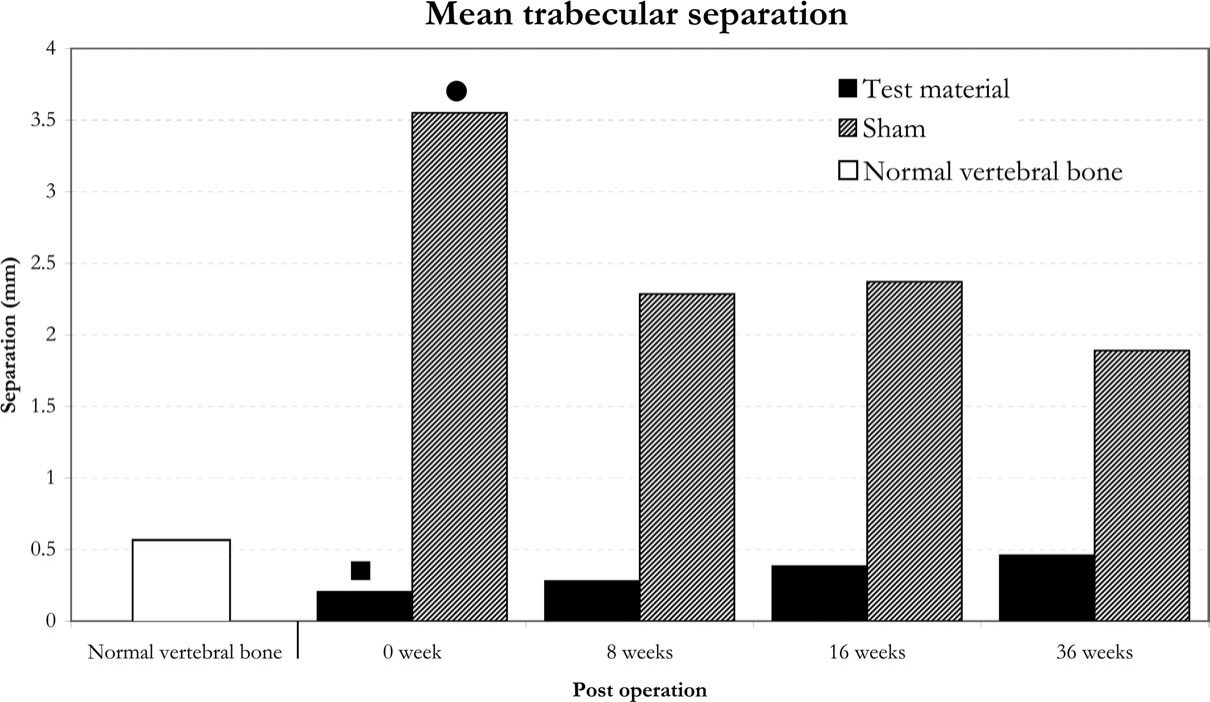
Mean trabecular separation for Test material and Sham sites, compared to that observed in normal vertebral trabecular bone. ▪ Low value at 0 week refers to test material filled sites and not trabecular separation. ● High value refers to empty sham sites.

**Figure 8:**
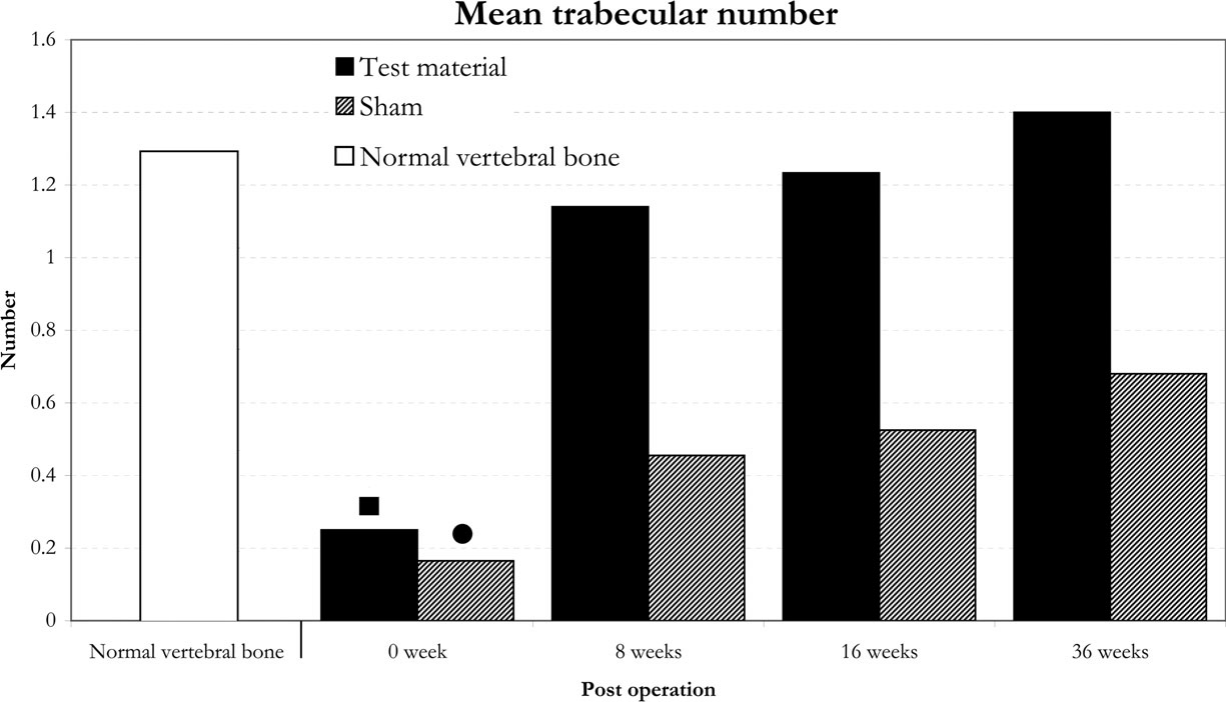
Mean trabecular number for Test material and Sham sites, compared to that observed in normal vertebral trabecular bone. ▪ Value at 0 week relates to test material-filled sites and not bone trabeculae. ● Value refers to empty sham sites.

Further comparative analysis of the two groups determined mean trabecular thickness (Figure 6), mean trabecular separation (Figure 7), and the trabecular number (Figure 8; a measure of the average number of trabeculae per unit length).

The mean trabecular thickness for both test material and sham groups indicates similar values between the groups from 8 to 36 weeks, although a gradual decline in the trabecular thickness was observed in the test material group. The mean trabecular separation showed a large difference in the sham group, indicating a more open trabecular structure than the test material and normal vertebral trabecular bone. A gradual increase in mean trabecular separation in the test material group reflected the gradual opening in the radiopaque tissue structure, approaching that of normal vertebral trabecular bone. Comparison of the mean trabecular number indicated the test material resulted in a large increase in trabeculae within the first 8 weeks, subsequently approaching that observed in normal vertebral trabecular bone. However, in the sham group, the increase in trabeculae was slower, and at each time point was much lower than that observed in normal vertebral trabecular bone. This indicated a reduced amount of trabecular bone structure in the sham group, confirming an incomplete return to normal bone architecture.

Although the number of samples in each group was small the differences observed were quite marked and followed a consistent pattern through the course of the study. The sham shows some repair of the defect with new trabecular bone but only around the edges of the cavity. PMMA fills the defect and extends into surrounding cancellous bone, with little change over the 36 weeks.

Similar to the PMMA the test material fills the defect at week 0. However, by 8 weeks there are signs of porosity and a formation of a trabecular structure in the defect. The volume of the test material declines over 36 weeks moving closer to a normal percentage of bone volume and density (g/cm^3^). Similarly the trabecular thickness, separation and number are close to normal values by 36 weeks.

### Histological analysis

**Figure 9:**
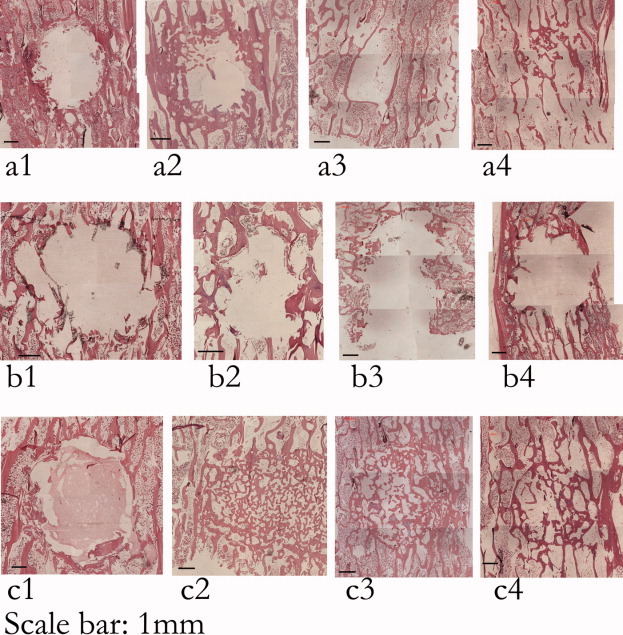
Paraffin histology, Mag x25, H&E Stain, Composite images. Sham procedure, (a1) Immediate, (a2) 8 weeks, (a3) 16 weeks, and (a4) 36 weeks post op. PMMA implanted vertebrae, (b1) Immediate, (b2) 8 weeks, (b3) 16 weeks, and (b4) 36 weeks post op. Test material implanted vertebrae, (c1) Immediate, (c2) 8 weeks, (c3) 16 weeks, and (c4) 36 weeks post op. [Color figure can be viewed in the online issue, which is available at wileyonlinelibrary.com.]

Histological observations were made on formalin-fixed, decalcified and paraffin wax-embedded bone samples (Figure 9). Additionally retrieved samples for resin embedding and sectioning were processed. Using this technique we were not able to make any observations that were not apparent on the decalcified, paraffin-embedded sections.

#### Sham group. 0 weeks

There was a circular defect containing fresh blood clot and a small amount of bone debris from the procedure. There was no surrounding bone tissue reaction; 8 weeks: A sclerotic rim of compact lamellar and woven bone had formed at the periphery of the hole. Fatty marrow had partially refilled the cavity; 16 weeks: The cavity had been filled in by trabecular woven and lamellar bone and accompanying fatty marrow. The original sclerotic rim of bone persisted; 36 weeks: Further resolution of the defect was evident. Evidence of the surgical procedure was the slightly thickened and disorientated bone trabeculae and the repopulation by fatty marrow.

#### PMMA group

**Figure 10:**
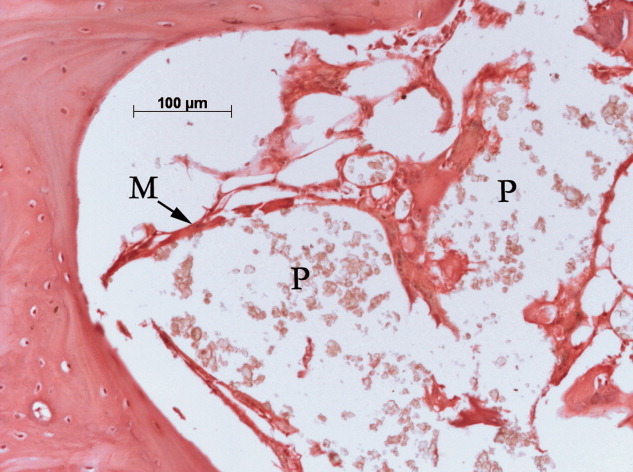
Paraffin histology, Mag ×200, H&E Stain. PMMA implanted vertebra. P, PMMA; M, fibrous membrane. [Color figure can be viewed in the online issue, which is available at wileyonlinelibrary.com.]

0 weeks: There was a circular defect containing PMMA residue and a small amount of bone debris. Focally, the PMMA could be seen to have extruded beyond the cavity into the surrounding marrow for up to 1 mm; 8 weeks: A sclerotic rim of compact lamellar and woven bone had formed at the periphery of the persistent cavity. This sclerotic rim appeared thicker than that seen in the sham procedure vertebrae at 8 weeks. The persistent PMMA had been broken up into loculi surrounded by a thin, fibrous membrane containing a macrophage reaction to the foreign material (Figure 10). There was no fibrosis, no acute inflammation, no lymphocytic reaction, and no granuloma formation in response to the PMMA at any time point. The PMMA had not been incorporated by the new, sclerotic bone. Around the cavity, there were zones of bone necrosis (empty osteocyte lacunae) defined within“cement” (reversal) lines within otherwise viable sclerotic trabeculae. These defined zones represent previous bone necrosis with subsequent new, still viable bony encasement; 16 weeks: The cavity persisted. Some sclerotic bone persisted, but this was less than that seen at 8 weeks. PMMA was still present, again as separate locules defined by thin fibrous membranes with a mild macrophage response. The PMMA had not been incorporated by the new, sclerotic bone; 36 weeks: The cavity persisted, with little change from the appearance at 16 weeks.

#### Drilled defect with subsequent test material grafting group

**Figure 11:**
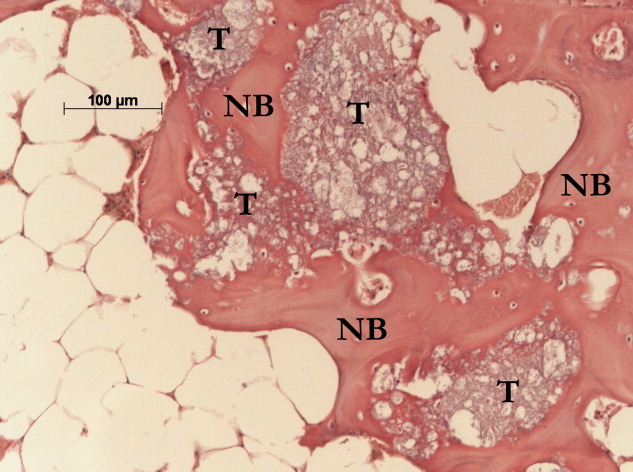
Paraffin histology, Mag ×200, H&E Stain. Test material implanted vertebra. T, test material; NB, new bone. [Color figure can be viewed in the online issue, which is available at wileyonlinelibrary.com.]

0 weeks: There was a circular defect containing the test material residue and a small amount of bone debris generated by the procedure. Focally, the test material was seen to have extruded beyond the cavity into the surrounding marrow for up to 1 mm; 8 weeks: The cavity had been completely filled in by new, woven bone and fatty marrow. The original site of the defect was still readily apparent as the new bone was densely packed and composed of thicker, shorter trabeculae which were much more interconnected than surrounding trabeculae. Furthermore, persistent, incorporated test material was readily apparent as bone-encased/incorporated refractile material (Figure 11). At this time point, most of the test material had either been resorbed or had become incorporated into bone. This became more so at subsequent time intervals. Where test material was not completely encased by bone, the cellular reaction to it was composed of macrophages (mononuclear or multinuclear). There was no fibrosis, no acute inflammation, no lymphocytic reaction, and no granuloma formation in response to the test material at any time point; 16 weeks: While the appearance was broadly similar to that seen at 8 weeks, there were some significant developments, *viz*. a degree of alignment of the new, cavity-filling bone trabeculae in parallel with the original trabeculae lying caudally and cranially to the cavity and, the more recently formed bone was mainly lamellar in nature. Again, test material was readily apparent as bone-encased/incorporated refractile material; 36 weeks: Three of the four vertebrae showed advanced resolution of the grafted cavity. The bone was lamellar in nature and the trabeculae were fewer in number with a thickness similar to that of the trabeculae seen away from the surgical site. Little test material remained. The marrow in the center of the lesion remained fatty rather than haemopoietic. However, new haemopoietic marrow was apparent centripetally at the periphery of the surgical site. The fourth sample was at a more immature stage, and was of a similar appearance to that seen at 16 weeks.

### Histomorphometric assessment

For the group implanted with PMMA, no new bone formation was seen within the area of the created void. Within the confidence limits of the macroscopically analysed histomorphometry 100% of the PMMA remained, although at microscopic analysis, a minimal breakdown of PMMA was evident at the periphery of the cement. In contrast, the majority of test material was resorbed within the first 8 weeks post implantation, with new calcified bone formation observed. The amount of new calcified bone formation at 8 weeks was comparable between the test material sites and the sham sites. However, at 16 and 36 weeks sacrifice points, the amount of new bone tissue was greater for the test materials sites than the sham procedure sites (Table 3).

**Table III:** Histomorphometric Assessment of Remaining Implant Materials and New Calcified Bone Tissue (Mean Values)

		Time			
Group		0 weeks	8 weeks	16 weeks	36 weeks
PMMA group	New bone formation (%)	0	0	0	0
	Material remaining (%)	100	100	100	100
Sham group	New bone formation (%)	0	48.9 ± 11.5	27.1 ± 17.1	26.3 ± 5.0
	Material remaining (%)	–	–	–	–
Test material group	New bone formation (%)	0	51.6 ± 4.5	51.3 ± 1.0	39.7 ± 4.8
	Material remaining (%)	100	1.6 ± 1.2	1.9 ± 1.2	1.2 ± 1.0

### Mechanical Testing

**Figure 12:**
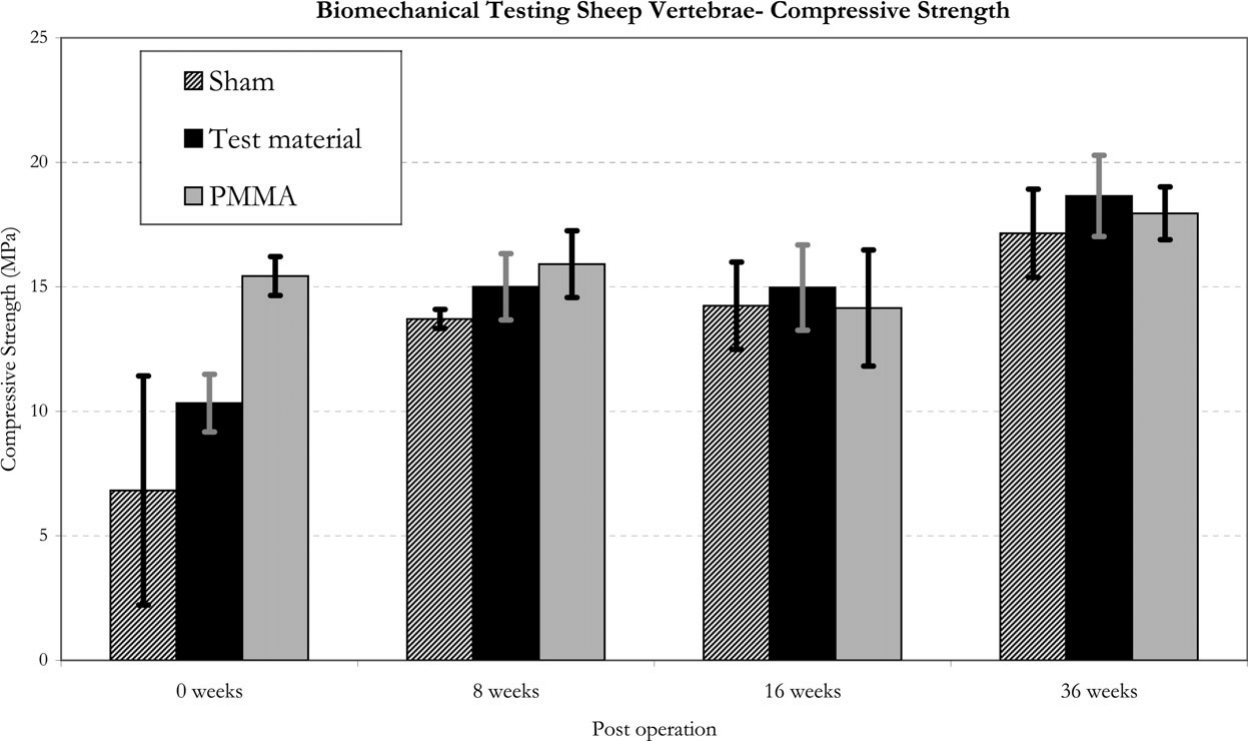
Compressive testing data. *22 comparison differences have statistical significance (Post hoc Tukey–Kramer test for multiple post hoc group comparisons, *p* < 0.05).

**Figure 13:**
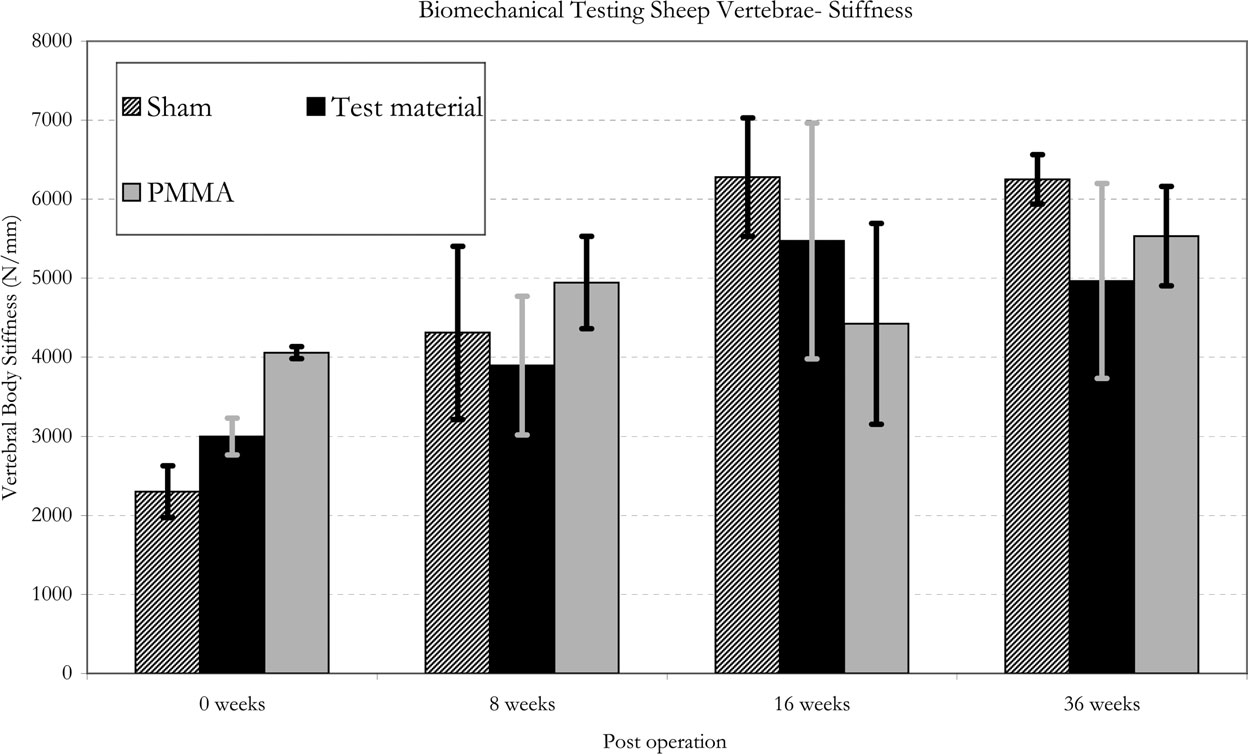
Stiffness testing data.

One vertebra implanted with test material was damaged during explantation from the 36 weeks postimplantation group and could not be used for mechanical testing (Tables 4 and 5, and Figures 12 and 13). The vertebrae implanted with PMMA had the highest compressive strength at 0 weeks. Vertebrae from sham and test material groups had a lower compressive strength at 0 weeks, with the sham group having the lowest compressive strength. No statistical significance was observed in vertebral body stiffness at this time point. At 8 weeks post implantation and all subsequent time points, there was no statistical difference between the compressive strength and stiffness between all three groups.

**Table IV:** Compressive Strength Results Summary [Compressive Strength (MPa)]

Group	Time				
	0 week	8 weeks	16 weeks	36 weeks
Empty group	6.8 ± 4.6^a^	13.7 ± 0.4	14.2 ± 1.7	17.1 ± 1.8
PMMA group	15.4 ± 0.8^a^	15.9 ± 1.3	14.1 ± 2.3	18.0 ± 1.1
Test material group	10.3 ± 1.2	15.0 ± 1.3	15.0 ± 1.7	18.7 ± 1.6

a Twenty-two comparison differences have statistical significance (Post hoc Tukey–Kramer test for multiple post hoc group comparisons, *p* < 0.05).

**V:** Vertebral Body Stiffness Results Summary [Vertebral Body Stiffness (N/mm)]

Group	Time				
	0 week	8 weeks	16 weeks	36 weeks
Empty group	2301 ± 328	4309 ± 1094	6278 ± 750	6251 ± 313
PMMA group	4056 ± 77	4943 ± 585	4423 ± 1269	5531 ± 630
Test material group	2999 ± 233	3895 ± 874	5471 ± 1493	4964 ± 1235

No statistical significance was observed in vertebral body stiffness.

## DISCUSSION

Excellent correlation was observed between the microCT and histology results. Comparison of the PMMA cement with the test material indicated a marked difference in both migration behaviour on implantation and bone regenerative response.

The microCT data show the advantages of the test material over PMMA or sham in terms of recovery towards a normal trabecular architecture. Although initially the test material completely fills the defect, over the course of a few weeks it is remodelled into a more normal bone structure. The test material has induced a repair process faster than no intervention (sham) and has the advantage over PMMA of being replaced by a normal bone structure.

Implant micromigration was observed at the first study time point of 0 weeks, for both materials on histological analysis. Using microCT analysis, extensive PMMA cement migration along the anteroposterior axis, towards the vertebral endplates was observed. The test material exhibited an improved host tissue response over the PMMA cement control. MicroCT and histological analysis indicated a marked osteoregenerative capacity of the test material when compared with the sham and the PMMA controls. The percentage of new bone formation was higher for the test material than the sham at 16 and 36 weeks post implantation, with the regeneration of new bone almost complete at 36 weeks postimplantation. For the sham group, regeneration of normal bone architecture was incomplete. For the PMMA group, new bone formation was limited to the periphery of the implant, at sites of previous bone necrosis.

The test material was associated with intense new bone formation within the cavity. As less test material remained at progressive time points, the bone appearance became more like that observed near to the surgical site. As the new bone and remaining material were removed by the apparent remodeling processes, the quantity and the appearance of the bone tissue indicated a return to normal bone architecture. The marrow in the lesion of the test material group remained predominantly fatty in nature, although at 36 weeks this was being replaced at the periphery of the implant site by new haemopoietic marrow. Histological analysis found that very little test material remained at 8 weeks post implantation. This can be understood when considering material composition. Fifty percent of the test material is calcium sulfate which will be resorbed *in-vivo* by dissolution within 12 weeks.^13^ Recent research has attempted to further determine the mechanism by which calcium sulfate supports bone growth. The implantation of calcium sulfate in a series of *in vitro* and *in vivo* studies (rabbit femur and tibia defect models) has been reported, determining the effects from a few days post implantation up to 16 weeks.^14^ In these studies, Ricci *et al.* reported that the calcium sulfate was observed to dissolve rapidly *in vitro* and *in vivo*, from the outer surface inward, and was observed to stimulate new bone formation. In most animal models calcium sulfate dissolved completely in as little as 4 weeks, leaving behind mineral deposits in the form of concentric rings in surrounding tissue. Histologically, these deposits were calcium phosphate deposits in the form of a precipitated carbonate apatite and were very similar to bone mineral in composition.^14^ The remaining β-TCP component of the test material would be expected to demonstrate a longer resorption profile.^15^, ^16^

PMMA implanted sites provide higher mechanical compressive strength and stiffness to the bone defect on setting, immediately after implantation, when compared to test material and sham groups. However, at 8–36 weeks, no statistical difference was observed between implant sites, suggesting the generation of adequate bone architecture from 8 weeks onwards. This restoration of strength in the test material and sham groups is presumed to be a result of sclerotic bone formation and the gradual restoration of the cortical bone at the point of surgical insult.

The required mechanical stability of an augmented vertebral body cannot be understated. Therefore, any alternative to PMMA in this demanding indication must demonstrate adequate strength and stability to prevent further vertebral collapse. Some success with calcium phosphate cements (CPCs) has been reported,^17^, ^18^ but observations regarding subsequent postoperative collapse,^19^, ^20^ indicate it's use is not recommended for vertebral augmentation as a result of the materials low resistance to flexural, tractive, and shear forces in comparison to PMMA.^21^

Indeed biomechanical evaluation of CPCs under a cyclic loading regime found evidence of fatigue.^22^

The relatively rapid resorption profile of the calcium sulfate phase may result in compromised mechanical strength in the immediate period post implantation, although some clinical success has been reported with calcium sulfate-based materials in vertebral augmentation.^9^, ^23^, ^24^ Previous research comparing calcium sulfate, PMMA, and CPCs has indicated that PMMA augmented vertebrae had the highest biomechanical strength and stiffness at 2, 12, and 24 weeks post implantation.^25^

The study presented here had some limitations. The study was intended as a wide ranging evaluation of the test material, to characterise key analysis parameters using varied complementary analysis tools. However, due to the limited animal number, the ability of the study to definitively investigate each method by statistical means was limited. Any further comparative study with the test material should be performed with a statistical population to expand on the observations noted in the presented research. Also, to fully understand the mechanical implications for the material resorption and transition to new bone formation observed with the test material, additional study is required focussing on the interim time frames from 0 to 8 weeks post implantation. This additional data will determine the effect of test material resorption on compressive strength of the vertebrae, which is of clinical importance for its consideration as an implant material. Also, the mechanical testing employed in this study did not incorporate fatigue testing, which is important to determine structural integrity in cyclically loaded environments. A further limitation to the study was the use of a surgical technique that did not reflect the transpedicular surgical approach typically employed in vertebral augmentation procedures. The model employed is therefore a bone defect model and not a vertebral compressive fracture model.

## CONCLUSIONS

The bone regenerative capacity of the test material was clearly demonstrated in this sheep vertebral bone defect model. Extensive new bone formation was observed at the early 8 weeks post implantation time point, in addition to resorption of the test material and the integration of residual graft into new bone tissue. The further resorption of the test material was demonstrated at the later study time points, and the return of the implant site to normal bone architecture was observed. The test material requires further investigation to establish its bone forming potential and the mechanical strength of the filled defect at early time points from 0 to 8 weeks post implantation.
